# Integration of Cypoviruses into polyhedrin matrix†

**DOI:** 10.1039/d3na00393k

**Published:** 2023-07-16

**Authors:** Olga V. Konevtsova, Ivan Yu. Golushko, Rudolf Podgornik, Sergei B. Rochal

**Affiliations:** a Physics Faculty, Southern Federal University Rostov-on-Don Russia rochal_s@yahoo.fr; b School of Physical Sciences and Kavli Institute for Theoretical Sciences, University of Chinese Academy of Sciences Beijing 100049 China podgornikrudolf@ucas.ac.cn; c CAS Key Laboratory of Soft Matter Physics, Institute of Physics, Chinese Academy of Sciences Beijing 100190 China; d Wenzhou Institute of the University of Chinese Academy of Sciences Wenzhou Zhejiang 325000 China

## Abstract

Unlike in other viruses, in Cypoviruses the genome is doubly protected since their icosahedral capsids are embedded into a perfect polyhedrin crystal. Current experimental methods cannot resolve the resulting interface structure and we propose a symmetry-based approach to predict it. We reveal a remarkable match between the surfaces of Cypovirus and the outer polyhedrin matrix. The match arises due to the preservation of the common tetragonal symmetry, allowing perfect contacts of polyhedrin trimers with VP1 and VP5 capsid proteins. We highlight a crucial role of the VP5 proteins in embedding the Cypovirus into the polyhedrin matrix and discuss the relationship between the nucleoside triphosphatase activity of the proteins and their role in the superstructure formation. Additionally, we propose an electrostatic mechanism that drives the viral superstructure disassembly occurring in the alkaline environment of the insect intestines. Our study may underpin novel strategies for engineering proteinaceous nanocontainers in diverse biotechnological and chemical applications.

## Abstract 2

Based on symmetry arguments we predict the architecture of the Cypovirus superstructure and propose an electrostatic mechanism for its disassembly in alkaline environment.

## Graphical abstract

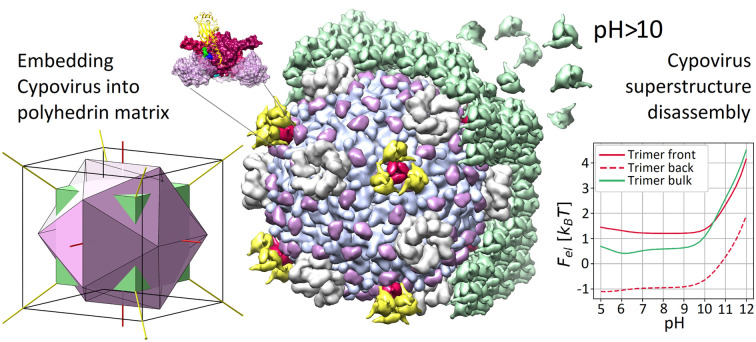

## Introduction

Cytoplasmic Polyhedrosis Viruses (CPVs) infect many insect species (predominantly Lepidoptera).^1^ CPV infected insects that were found fossilized in amber provide evidence that these viruses have existed for at least 100 million years.^2^ Cypovirus is a genus of one of the largest RNA viral families, *Reoviridae*. Unlike in most Reoviruses, which generally have a multilayered capsid consisting of several concentric protein shells, in CPVs, the genome is enclosed in a single capsid shell. Another distinctive feature of this genus is a unique mechanism for transporting and protecting their genetic material, which involves the formation of cubic viral superstructures that are visible in an optical microscope and contain multiple capsids surrounded by the matrix of polyhedrin molecules. The latter are encoded by the viral genome and self-assemble in the cytoplasm of infected cells, forming robust polyhedra with regular faceting and periodic structure, in which the preliminary assembled virions are embedded during crystallogenesis.^3^

Due to their high density and mechanical strength, the resulting composite superstructures allow viruses to survive during periods unfavourable for reproduction, for example, in winter, when insects are not active. These superstructures are resistant to extreme biochemical conditions, including concentrated acids, detergents, and solvents, but are quite sensitive to alkaline pH,^4^ an important facet of the infection process by this virus. CPV polyhedra enter the insect body *via* the fecal-oral route, and later get into the strongly alkaline environment of the larvae midgut, where they disassemble and release virions. Then they penetrate the insect cells and trigger the production of new viral crystals, which are excreted by the insect for the rest of its life.^3^

There are more than 20 types of Cypoviruses and the characteristic volume of their superstructures varies from 10^−3^ to 10^3^ μm^3^, depending on the type.^5^ For example, CPV17 superstructures usually contain only one virion and are typically 100 nm across,^6^ while polyhedra of most other Cypoviruses can contain several thousand viral particles each.^7–9^ In 2007, synchrotron X-ray diffraction was first applied to elucidate the structures of such large polyhedra,^4^ while the superstructure formed by CPV17 was analysed 8 years later.^5^

**Fig. 1:**
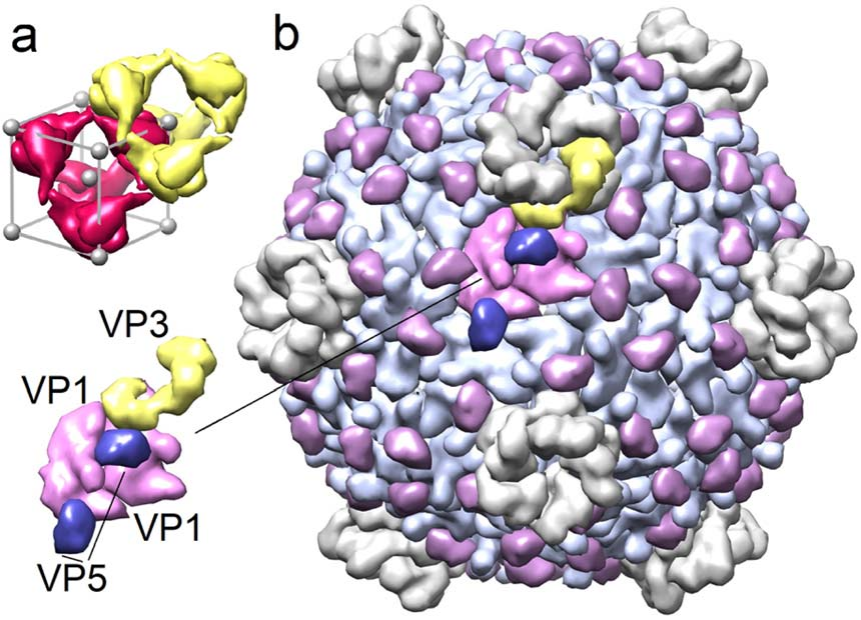
Structures of polyhedrin crystal and Cypovirus capsid. (a) Two polyhedrin tetrahedrons with the centers at the nodes of body centered cubic lattice. (b) Cypovirus capsid and its asymmetric structural unit consisting of five structural proteins. Sixty such units form the entire shell.

Despite significant differences in the amino acid composition of the polyhedrin proteins of different CPVs, they form nearly the same body-centred cubic crystal structures with a slightly varying periodicity and exhibit I23 chiral symmetry. The 12 proteins forming the primitive cell can be represented as a regular tetrahedron with centres of four protein trimers located at tetrahedron vertices.^4^Fig. 1a shows two such tetrahedrons surrounding the nodes of body centred cubic polyhedrin lattice.

Polyhedrin proteins are encoded by one of the 10 RNA segments of the Cypovirus genome.^13,14^ Three more segments encode the main structural proteins of the CPV viral capsid VP1, VP3 and VP5.^13^ 120 VP1 proteins are organized into 60 dimers that form the icosahedral shell, with VP3 and VP5 proteins located on its outer surface. VP3 are arranged around 5-fold symmetry axes of the capsid, forming pentameric turrets,^13^ while 120 VP5 proteins are located at the pair junctions of neighboring VP1 proteins (Fig. 1b).

Although the structures of various CPV capsid types and the corresponding polyhedrin crystals have been studied in detail, the mechanisms controlling the self-assembly and disassembly of the viral polyhedra are still poorly understood. Moreover, currently there are no structural models describing the geometry of the capsid incorporation into the polyhedrin matrix. In fact, the contact regions between the CPV capsid and the polyhedrin molecules have also not been established yet, due to the lack of high resolution microscopy data.^3^ The initial hypothesis^10^ that polyhedrin molecules attach to the VP3 capsid proteins, forming so-called turrets on the 5-fold axes, has been actually disproved.^3,11,12^ At the same time, it is clear that the contact regions and the nature of the interaction between viral capsid and polyhedrin molecules play a key role in the self-assembly and disassembly of the CPV superstructures. Studying the interactions between the polyhedrin proteins and the viral shell surface may be additionally important for the development of new biotechnological applications, based on the encapsulation of molecules or molecular complexes of interest in a polyhedrin matrix, that can release its content at a controlled pH level.

Thus, there is a clearly identifiable gap in our knowledge of how the icosahedral shell is incorporated into the outer polyhedrin crystal matrix at the level of individual proteins and contacts between them. In what follows, we propose a structural model of the resulting complex assembly, based on general symmetry principles and group theory, and show how the removal of a certain number of trimers from the polyhedrin crystal leads to the formation of a cage with *T* symmetry, which is a subgroup of the CPV capsid symmetry group I. The virus, being in the center of this cavity, forms close contacts (with a gap smaller than the characteristic size of a water molecule of 2.8 Å) with the rest of the crystal, such that the more important contacts are located along the four common 3-fold axes of the subgroup *T*. Based on the features of the resulting superstructure organization, we clarify the role of the VP5 capsid protein. Also, within the framework of a simple electrostatic model based on the protein charge distribution, we unravel the disassembly mechanism of the viral shell – polyhedrin matrix superstructure, and the release of embedded virions in the highly alkaline environment of the insect larvae midgut.

## Results and discussion

### Structural model

**Fig. 2:**
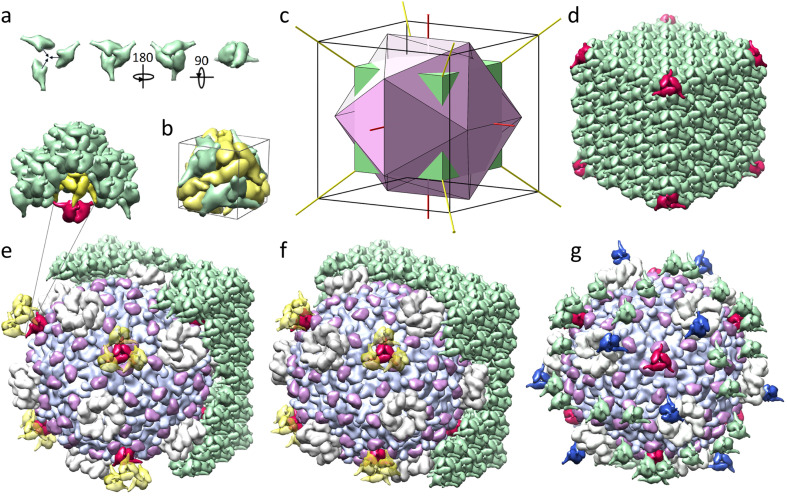
Embedding of the Cypovirus capsid into the polyhedrin crystal matrix. (a) Polyhedrin trimer shown in different orientations. (b) Primitive cubic cell of the polyhedrin crystal containing eight trimers. More convex frontal sides of four green trimers are facing the cell center; four yellow trimers are facing outward. (c) Icosahedron inscribed into a cube and superimposed with a smaller cube. Edges of the cubes are related as 2 : 3. Common rotational axes of the groups *T* and *I* are shown in red and yellow. (d) A fragment of the polyhedrin crystal formed by 4 × 4 × 4 primitive cells. Eight trimers (shown in red) in the vertices of the fragment are the only ones located outside of the virus capsid and potentially not excluded upon virus embedding. (e and f) Two variants of the capsid embedding into the polyhedrin cage (see the main text). All trimers of the 3 × 6 × 6 cluster that are not excluded upon virus embedding, are shown in green. Remaining trimers (shown in red) belong to the 4 × 4 × 4 fragment and occupy 3-fold axes of the superstructure. Triplets of the closest neighbors of the red trimers are shown in yellow. The insert in panel e shows the cavity behind the trimers facing the virus in the depicted embedding variant. VP1 and VP5 proteins are shown in light purple and purple, respectively, while 5-fold turrets assembled from VP3 are shown grey. (g) The viral surface and trimers contacting it in the second embedding variant showing the trimers that have contact area exceeding 260 Å^2^. Blue trimers are in contact with VP3; green trimers are in contact with VP5.

Although viral polyhedra can be easily observed even with conventional light microscopy, they are too small for standard methods of crystal structure determination, and the structure of the viral polyhedrin was first deciphered only in 2007 for CPV1.^4^ The polyhedrin crystal matrix is constructed of compact trimers,^3,4^ shown in different orientations in Fig. 2a (all images of protein structures were obtained with UCSF Chimera).^15^ Four such trimers, colored in green in Fig. 2b, form a tetrahedral cluster. The more convex side of the trimers facing the cluster center is hereinafter referred to as the frontal side. The trimer mass centers lie on the 3-fold symmetry axes of this cluster. The yellow trimers shown in Fig. 2b are translationally equivalent to the green ones with respect to the translations (±*a*, ±*a*, ±*a*)/2. They make contacts with each other through alpha helices, protruding perpendicularly to the trimer 3-fold axis. The mass centers of yellow trimers are located further from the common center than the green ones and thus form a larger tetrahedron. Two such tetrahedrons are shown in Fig. 1a. Small (inner) and large (outer) tetrahedrons form a primitive unit cell shown in Fig. 2b. The cell contains eight trimers and has a size of 101.7–106.1 Å depending on the Cypovirus type. The cells are organized into a three-dimensional body-centered cubic lattice of polyhedrin crystals that have a cubic shape and a micrometer size.^4^ It is interesting to note that even the simplest CPV17 superstructure, containing only one icosahedral virion, also exhibits a relatively regular cubic shape.^6,16^

It is well known that the presence of impurities affects the average lattice parameters and faceting of a crystal. In viral polyhedra, while embedded icosahedral virions act as such impurities, even a relatively large concentration of virions neither induces a distortion of the polyhedra faceting, nor a significant change in the parameters of the crystal lattice,^3,4^ suggesting the existence of a commensurability (*i.e.* a good match) between the crystal structure of polyhedrin and the icosahedral capsid of the virion.

To find this commensurability, one should recall that an icosahedron can easily be inscribed into a cube (Fig. 2c), and that the symmetry group *T* of the tetrahedral polyhedrin cluster is a subgroup of the group *I* of icosahedral CPV capsid. However, the primitive cell of polyhedrin crystal is much smaller in size than the virus itself, for example, *Bombyx mori* CPV1 capsid is approximately 660 Å in diameter (excluding the fivefold turrets).^13^ In order to construct a detailed model of the CPV superstructure, we used CPV1 capsid (3JAY) and its polyhedrin (5GQL) as the most structurally studied protein assemblies resolved at 3.00 Å and 1.78 Å, respectively. Here and below, IDs of protein structures are given according to the PDB.^17^ The period *a* of this polyhedrin lattice is 103.33 Å, and the maximum size of the capsid along its 2-fold axis is 662.49 Å, which means that the viral capsid is approximately equal in size to a cluster of a 6 × 6 × 6 primitive cells.

Let us note that the icosahedral symmetry *I* of the viral shell is crystallographically incompatible with the translational order of the polyhedrin, whose orientational order is characterized by the group *T* corresponding to all rotations of the tetrahedron. Being the maximal common subgroup of symmetry groups of the matrix and the shell, the group *T* should provide the best match between the surfaces of the capsid and the polyhedrin cage. The value 6*a* ≈ 620 Å, however, differs quite significantly from the capsid size, but if we consider dimensions of polyhedrin molecules forming the 6 × 6 × 6 cluster of body-centered cubic cells and align this cluster with the virus capsid, so that their centers and common 2-fold and 3-fold axes coincide, matching between sizes of the cluster and the capsid becomes almost perfect. The distance along the 2-fold axes between the most protruding points of the polyhedrin tetrahedral cluster is 659 Å, which differs from the same distance in the capsid by less than 1%. This remarkable commensurability suggests that the virus is symmetrically embedded into the crystal by substituting most of the polyhedrin molecules in the considered 6 × 6 × 6 cluster. Our crystallographic analysis presented below lends further support to this model.

As can be seen from Fig. 2c, the smaller 4 × 4 × 4 cube extends beyond the icosahedron inscribed into the 6 × 6 × 6 cube, which means that the trimers located in the vertices of the 4 × 4 × 4 polyhedrin cluster (see Fig. 2d) could reside on the surface of the virion, occupying the common 3-fold axes of the resulting superstructure. For example, for axis (111), such trimers would have center of mass coordinates (1.5*a* + *x*_1_)(1,1,1) and (−1.5*a* + *x*_2_)(1,1,1) where *x*_1_ + *x*_2_ = *a*/2 and for Bombyx mori CPV1 *x*_1_ = 30.494 Å and *x*_2_ = 21.170 Å. The second trimer, whose mass center is slightly closer to the common origin, faces the capsid with its frontal side, which could improve its contact with the surface of the virus shell. Upon embedding (Fig. 2e), it becomes obvious that the capsid should replace all polyhedrin molecules of the 4 × 4 × 4 cubic cluster, except for eight trimers shown in red in Fig. 2d (four facing the virus and four facing in the opposite direction), which require a more detailed analysis of their local environments.

Note that even though 90° rotations around the coordinate axis are not symmetry elements of the group *T*, they map this group onto itself (represents the external automorphism of the group). Since upon 90° rotations around the 2-fold axes, the polyhedrin lattice does not coincide with itself, there are two variants of embedding icosahedral virion into polyhedra, which result into two different superstructures with symmetry *T*.

In both variants of the embedding, we choose the axes of the coordinate system according to the polyhedrin PDB model 5GQL. Since the 5-fold axis of the capsid structure 3JAY coincides with the *z* axis, we rotate the capsid to align its 2-fold axes with the axes of the chosen coordinate system. In the first variant, one of 5-fold axes of the capsid lies along the vector (1,1,*t*) where *t* = √5 + 1 is the golden mean. In the second variant, the capsid is additionally rotated by 90° around the *z*-axis. Such a rotation keeps the icosahedron inscribed into the same cube and in terms of local environments is equivalent to turning the polyhedrin crystal by the same angle, but in the opposite direction. In two considered mutual orientations of the polyhedrin lattice and the virus capsid, eight remaining trimers of the 4 × 4 × 4 cubic cluster that also lie on the 3-fold axes of the capsid have four different possible local environments (two per orientation). In all of them, the polyhedrin trimer is surrounded by three VP5 proteins and located above the center of the VP1 proteins connected in a triplet (Fig. 2e and f).

Positioning of these eight polyhedrin trimers on the capsid surface suggests that their interaction with the virus could be pivotal for the embedding process. To analyze this hypothesis, we estimated the overlap between capsid proteins and the considered polyhedrin trimers. Using the effective noncovalent atomic radii^18^ we calculated the number of trimer atoms that are located too close to virus protein atoms (distance between atoms is smaller than the sum of their non-covalent radii). In the first mutual orientation of the capsid and polyhedrin matrix (Fig. 2e), trimers facing the virus have 3.97% of such positions, and trimers facing away from the virus, which are also slightly further away from the centre of the capsid, have no such “intersections” at all. The smallest distance between the atoms of the outward facing trimers and the atoms of the capsid is ∼7 Å, meaning that these trimers have no contacts with the capsid. In the second orientation (Fig. 2f), the percentages of such overlapping atoms in trimers are 1.74 and 0.4%, respectively.

Insignificant overlaps of proteins could be compensated for by their deformations during the embedding process. Since these deformations should be distributed between capsid proteins and polyhedrin trimers, their value should be smaller than the corresponding fractions of the overlapping atoms. Therefore, when modelling the cage, in which the virus is embedded, we allow a weak overlap of neighbouring proteins. To find trimers that should be excluded, we choose the limit values of overlaps to be 4 and 1.75% for the first and second orientations, correspondingly, so that all trimers located at the vertices of 4 × 4 × 4 polyhedrin cluster are not removed. Since viral polyhedra are known to self-assemble from pre-assembled trimers^3,4^ we excluded the entire trimer if even one of its proteins violated the limiting percentage of atomic overlap.

This algorithm leads to the exclusion of 788 and 836 polyhedrin trimers in the first and second considered orientations, respectively; 48 and 96 of these trimers are excluded from the regions outside of the 6 × 6 × 6 cluster. In both cavities, among all remaining trimers, mass centres of the eight trimers belonging to the vertices of the 4 × 4 × 4 cluster turn out to be the closest to the capsid centre. Importantly, in the first capsid orientation, the nearest neighbours of the four trimers facing the capsid are excluded, which creates cavities behind them (Fig. 2e). These trimers, while having substantial contacts with the virus, are attached to the bulk polyhedrin crystal only *via* small contacts between protruding alpha helices (insert in Fig. 2e), and therefore the first orientation not only requires protein deformations that are more than twice as large (3.97 *vs.* 1.74% of overlapping atoms), but also leads to a less robust fixation of the virus in the cavity than the second orientation, suggesting that the second variant of embedding (ESI Movie 1†) is much more plausible for the superstructure formation.

### Peculiarities of the interface structure and roles of VP5 proteins

**Fig. 3:**
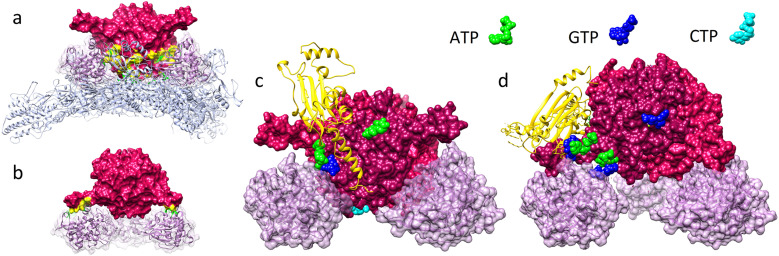
Arrangement of the Cypovirus superstructure in the vicinity of the 3-fold axes. (a and b) Contact regions (highlighted in yellow) between trimers occupying 3-fold axes and capsid proteins. (c and d) Localization of NTP molecules in the vicinity of VP5 proteins. In panels (a and c), trimer faces the virus (also see ESI Movie 2†) and in panels (b and d), trimer faces the opposite direction. ATP in the middle of panel (c), located far from VP5 proteins, is in contact with them in the opposite trimer orientation shown in panel (d). The same applies for GTP in panel (d).

Let us characterize more precisely the contacts between the viral surface and the polyhedrin cage. Conventionally, regions of two proteins are considered to be in contact if there is no place for water molecule to fit in between. Contacts between proteins can be characterized by buried surface area (BSA) value,^19^ which is equal to the decrease in solvent accessible surface (SAS) area when two proteins are brought into the contact with each other. As our calculations show, in the second orientation with the overlap value limited to 1.75%, the four closest trimers facing the capsid have BSA = 6400 Å^2^ each, with approximately 4000 Å^2^ coming from contacts with VP5 proteins (Fig. 3a). The other four trimers, facing the opposite direction, form contacts exclusively with VP5 proteins having BSA = 1550 Å^2^ per trimer.

Note that these trimers are not the only ones contacting the capsid. The total BSA associated with the superstructure formation equals ∼76 540 Å^2^ with ∼41.5% given by the trimers occupying 3-fold axes and ∼58.5% given by other trimers occupying eleven 12-fold position of the group *T*, five of which are shown in Fig. 2g in green and blue. These positions have contact areas larger than 260 Å^2^ per trimer. In the first orientation, for the same overlap limit of 1.75%, there are only five analogous general crystallographic positions occupied with trimmers and the total BSA of the resulting superstructure equals ∼20 270 Å^2^, which is almost four times smaller. For maximum overlaps of 1 and 3% the picture does not change qualitatively, and in both cases, the second orientation has more trimers in contact with the capsid and a larger total BSA, which once again confirms that this embedding variant should be implemented *in vivo*.

According to the experimental study,^12^ polyhedrin can bind to structural proteins VP2, VP4 and VP5, interacting also with individual domains of the VP1 structural protein. VP2 and VP4 proteins are attached to the inner part of the capsid,^20–22^ and therefore they cannot participate in the capsid embedding into the polyhedrin matrix. Regions of the VP1 protein, which were experimentally shown to interact with polyhedrin molecules, are also in contact with the polyhedrin proteins in our model of the superstructure, but the estimates of the contact areas show that in comparison with VP1 proteins, the role of VP5 proteins in the capsid embedding is more significant.

For a long time, it was believed that VP5 proteins simply stabilize the capsid, until it became clear that the VP5 proteins are also involved in the RNA chaperone^23^ and the nucleoside triphosphatase (NTPase) activities.^24^ While today, we know that VP5 proteins contribute to the hydrolysis of all types of NTP and dNTP molecules,^24^ it remains unclear why these proteins exhibit NTPase activity.^24^ In addition, for reasons still unknown, many CPV superstructures contain ATP, CTP and GTP nucleotides, attached to polyhedrin molecules at specific locations^4,14^ and comparison with experimental data from ref. 4 shows that GTP nucleotides attach to approximately the same locations on trimer surface, where in our model the trimers occupying 3-fold axes and facing the virus are in contact with surrounding VP5 proteins (Fig. 3c). As Fig. 3c reveals, VP5 proteins also come in contact with ATP nucleotides, but these nucleotides are associated with three polyhedrin trimers neighboring the one facing the virus and highlighted in red. In Fig. 3c, one polyhedrin molecule belonging to such a neighboring trimer is shown with yellow ribbons, forming a small contact with VP5 (BSA ≈ 480 Å^2^).

Similar localization of NTP nucleotides can be seen in the vicinity of contacts between VP5 proteins and trimers occupying the 3-fold axes and facing away from the virus surface. Each of them together with three neighboring trimers (BSA ≈ 140 Å^2^ per trimer) also appears to be attached to VP5 *via* ATP + GTP nucleotide complex (Fig. 3d). We performed the same analysis for all other polyhedrin trimers contributing to the BSA of the superstructure formation and found that trimers in seven of eleven general crystallographic positions have contacts with VP5 proteins. In five of these positions (highlighted in Fig. 2f and g with yellow and green, respectively), NTF nucleotides are located in the contact regions. Overall, trimers with NTF molecules contacting VP5 proteins provide the major contribution (∼50 000 Å^2^) to the total BSA between the virus and the polyhedrin cage.

Thus, the ability of VP5 proteins to hydrolyze NTP molecules (which peaks at pH = 8 and then decreases approximately two times at pH = 10)^24^ seems to be associated with the ability of Cypovirus to attach/detach polyhedrin trimers in an alkaline environment. Note also that the CTP molecules at the front side of the polyhedrin trimer^4^ are located close to the VP1 capsid proteins (Fig. 3c), but NTPase activity of the VP1, or lack thereof is yet to be demonstrated. In this context we note that the locations of VP1 proteins and the CTP molecules are not as close as the ones of ATP/GTP and VP5.

The apparent multifunctionality of VP5 proteins is not surprising, since, for example, VP3 also performs multiple other functions: it serves as a capping enzyme, a signaling sensor, and forms channels for mRNA release on the 5-fold axes of the CPV capsid.^10,13,20,21,25,26^

We have developed a detailed structural model describing the interface between the CPV surface and polyhedrin cage; in the next section, we propose and discuss the electrostatic mechanism of the considered superstructure disassembly, which occurs in an alkaline environment and is essential for the CPV infection process.

### Electrostatic mechanism of the virus superstructure disassembly

Electrostatic forces play a key role in many biological processes involving protein molecules.^27,28^ Proteins in the bathing solution almost always, except at the isoelectric point, carry a non-zero electric charge,^29^ a consequence of ionizable amino acid (AA) residues. The degree of amino acid ionization depends on the properties of the bathing solution, among which the pH and electrolyte concentration are the most important ones. At a certain pH levels the total charge of a protein can reach several tens of elementary charges^30^ and can be of either sign. Changes in pH of the bathing solution can lead specifically to substantial changes in electrostatic charge of the viral proteins^31^ that can in its turn drive global structural rearrangements of protein assemblies.^32,33^ Motivated by the experimental findings^4,14,34^ that disassembly of Cypovirus polyhedra and release of the embedded virions are strongly dependent on the acidity of the surrounding solution, we now examine the molecular details of how the increase in pH affects interactions between viral proteins and polyhedrin trimers.

We emphasize that the main goal of our analysis is not to develop a precise quantitative electrostatic model of the system, but to identify a key mechanism driving the disassembly process. Therefore, we use a coarse-grained model that simplifies the detailed electrostatics by considering a point-charge model of ionizable AAs. We assume that only those AAs that are exposed to the aqueous solution can dissociate. Conventionally, AA is considered exposed only if a substantial part of its surface is in contact with the buffer.^35–37^ To find these AAs contributing to the net protein charge, following ref. 35 and 38, we calculate the solvent accessible surface area for each ionizable AA in the structure and compare it with the area of the same AA in the reference state. A relation between these areas, called the relative solvent accessibility (RSA), characterizes the degree of exposure of the AA residue to the solution, and in general depends on the assumed threshold value of RSA separating exposed and buried residues, as well as on the AA conformational state taken as the reference one.^34–37^ In our semi-qualitative model, we choose RSA threshold to be 25% and use the conformations of AAs in the considered proteins as the reference ones.

**Fig. 4:**
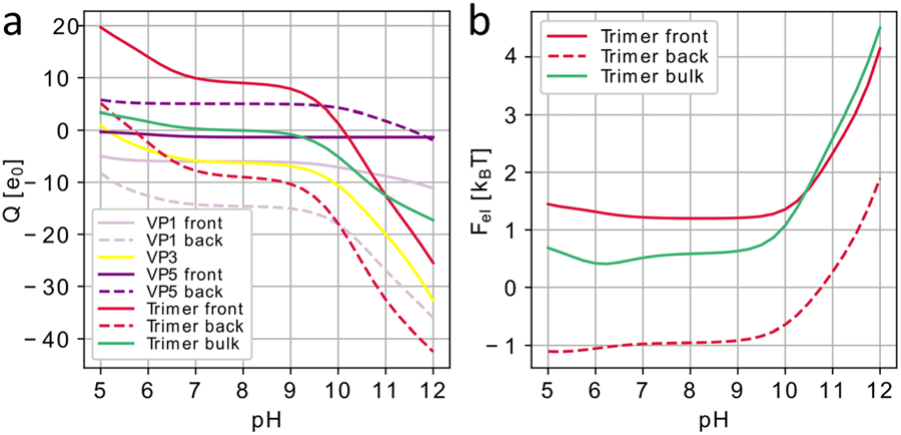
Electrostatic effects associated with Cypovirus superstructure disassembly. (a) Charges of CPV1 capsid (3JAY) proteins and polyhedrin (5GQL) trimers occupying positions at 3-fold axes of the capsid expressed in the elementary charge units *e*_0_ as a function of the pH level. Data for the proteins in the vicinity of the trimers facing virus capsid (labeled “front”) is shown with solid lines and data for the proteins in the vicinity of the trimers facing the opposite direction (labeled “back”) are shown with dotted lines. (b) Effective electrostatic energy of polyhedrin crystal normalized per trimer as a function of pH (green) and electrostatic interaction energy between Cypovirus capsid and polyhedrin trimers, both in the units of *k*_B_*T*.

After finding all AAs that are exposed to the solution and thus can be dissociated, we use the Henderson–Hasselbalch (Langmuir) dissociation isotherm^39,40^ to obtain their partial charges, disregarding the details of the electrostatic self-interaction. The presence of free ions in the bathing electrolyte solution not only modifies the ionization of AAs but also leads to electrostatic screening of their charges. The energy of the electrostatic interaction between proteins of interest can then be calculated within the Debye–Hückel approximation as a sum of energies of pairwise screened Coulomb interactions between their residues (see Methods). Standardly the screening length under physiological conditions can be estimated to be *λ*_D_ ≈ 1 nm.^32,33,41^ First, let us discuss how the trimer charge and electrostatic energy of CPV1 polyhedrin crystal (5GQL) change with the increasing pH. Fig. 4a shows that a single polyhedrin trimer in the bulk crystal has an isoelectric point at pH ≈ 8 and rapidly gains negative electrostatic charge with the increase of pH for pH > 9 (green curve). Regardless of the possible parameters of the electrostatic model, the rapid growth of the trimer charge with the increase of pH should lead to the increase of electrostatic (free) energy of the system that would subsequently destabilize it. This scenario fully agrees with the observations that polyhedrin crystals dissociate in solvents with pH > 10.5.^4^

The normalized electrostatic energy of the polyhedrin crystal as a function of pH is shown in Fig. 4b (green plot) and given in units of *k*_B_*T* normalized to the total number of trimers, so it can be interpreted as the interaction energy of one (central) trimer with all other equivalent trimers. Since the polyhedrin crystal is assembled from unit trimers, we did not consider the electrostatic interaction energy between the proteins within the trimer. The sum of pair interaction energies between the trimer and its neighbours quickly converges due to the exponential decay of the considered potential with the distance (see Methods), and for screening lengths at physiological conditions, only trimers inside a sphere with the radius of two-unit cells surrounding the central trimer give a meaningful contribution to the total energy. As is obvious from Fig. 4b, the normalized electrostatic energy undergoes a drastic increase in the region pH > 10, which should eventually lead to the disruption of the crystal.

Disassembly of the polyhedra, however, does not necessarily imply detachment of individual polyhedrin trimers from the virus, and we examined also how the energy of the electrostatic interaction between the Cypovirus capsid (3JAY) and the eight closest polyhedrin trimers changes within the framework of the proposed superstructure model (Fig. 2f). Fig. 4 shows polyhedrin trimer charges (panel a) and electrostatic energies of interaction with the virus (panel b); red solid lines correspond to trimers facing the virus and dashed lines correspond to those with the opposite orientation. In both cases, a more alkaline environment leads to a higher negative charge (panel a). The differences in the functions describing the trimer charges of the inward and outward facing trimers are due to differences in the residues that are exposed to the buffer solution. The graphs in Fig. 4b show that the normalized electrostatic energy increases with increasing pH, making attachment of the trimers to the virus capsid less and less energetically favourable. This fact should eventually lead to their detachment from the shell, since with increasing pH, the virus capsid also becomes negatively charged, as can be seen from the graphs in Fig. 4a, showing charges of the structural proteins. Note that due to the embedding of the virus into the polyhedra and a corresponding decrease in symmetry, *I* → *T*, the structural proteins on the opposite sides of the virus around the 3-fold axis become symmetrically non-equivalent and thus acquire different charges. In particular, VP5 proteins contacting the front side of polyhedrin trimers bare small negative charges whereas their counterparts contacting the back of the trimers are positively charged. Fig. 4a also shows that at pH ≈ 10 the trimer facing the capsid becomes negatively charged and begins to repel from nearest negatively charged VP5 capsid proteins. This again emphasizes the special role of VP5 proteins in the formation of the superstructure, since virions are released from the polyhedrin matrix at pH > 10.5.^3,14^

The proposed model unveils the electrostatic mechanism driving disassembly of the CPV superstructures in alkaline medium and shows a good qualitative agreement with the experimental data. However, even though the approach we used should describe electrostatic charges of proteins as functions of pH relatively accurately,^35^ the electrostatic interaction energy between proteins in a more complex and realistic model could be significantly higher, especially in polyhedrin crystals. Here, within the framework of the developed model, we took the same values of the effective screening length *λ*_D_ and permittivity *ε* (see Methods) that we previously used to model other protein systems.^32,33^ More accurate estimations can be obtained by solving complex nonlinear equations describing the distribution of electrostatic potential in the nonhomogeneous dielectric medium.^42^ Even without finding explicit solution of this problem one can assume that due to the high density of the polyhedrin crystal, the effective permittivity could be substantially lower and the effective screening length substantially higher than our initial estimate.^43^ As is easy to show, these changes can only increase the electrostatic repulsion between the proteins in the alkaline solution.

## Conclusions

Cypoviruses have a unique mechanism for protecting and delivering their genome. Once inside a cell, in addition to the expression of capsid proteins, the virus genome induces an overexpression of the polyhedrin protein that then self-assembles into dense molecular crystals with embedded Cypovirus capsids inside. Although Cypoviruses have been actively studied over the last 15 years, and structures of capsids and polyhedrins of multiple types of Cypoviruses have been determined with high resolution, a detailed explanation for how the icosahedral viral capsid is embedded into the tetragonal crystal structure at the level of individual proteins and their contacts was lacking.

Our model is filling this gap. We used a well-known crystallographic notion that two structures, *e.g.* a film and a substrate, can form a well-aligned and robust superstructure only if they have a common symmetry subgroup, which determines the architecture and symmetry of the interface between the two structures. Since in the considered case, the capsid has the point symmetry *I* and the outer polyhedrin crystal has the spatial cubic symmetry *I*23, the maximum common subgroup is the point group *T* of all tetrahedron rotations. Based on this idea, we position the virus with respect to the polyhedrin lattice so that the capsid center coincides with one of the lattice nodes and its 2-fold and 3-fold axes are aligned with those of the group *T*, which is the local symmetry group of this node. There are two variants of such an embedding, and one of them results in an almost perfect match between the surface of the viral capsid and the surface of the respective cavity in the polyhedrin crystal, formed after the exclusion of 12*N* polyhedrin molecules, where *N* = 209. A critical role in embedding the Cypovirus into the polyhedrin matrix is played by VP5 capsid proteins,^21^ which almost ideally match the polyhedrin trimers. By analysing the regions of contact between the proteins we proposed a relation between the nucleoside triphosphatase activity of VP5 proteins and their role in the formation of the superstructure. Our analysis also revealed the regions, where ATP, CTP and GTP nucleotides could attach to the capsid surface and, in particular, to VP5 proteins, which is another important finding based on geometric considerations.

The structural model developed allowed us to propose an electrostatic mechanism that controls the disassembly of the superstructure in the alkaline environment of infected insect larvae midgut. Our semi-quantitative electrostatic model unambiguously shows that high pH induces changes in the charge dissociation of solvent-exposed AA residues enhancing the negative charges of polyhedrin molecules. The resulting protein charging on the one hand induces a sharp increase in the (free) electrostatic energy of polyhedrin crystals, which triggers their disassembly, and on the other weakens the binding of polyhedrin trimers to the virus as the trimers start to be electrostatically repelled from the capsid surface. Overall, our model clearly highlights the crucial role of the electrostatics in the onset of the CPV infection process.

In order to assess the possible applications of our findings we note that various extrinsic proteins, including human ones, can be immobilized in artificial viral polyhedra^44^ serving as nanocontainers that can be used for a sustained release of cytokines and control of cell proliferation and differentiation.^44^ These nanocontainers stabilize embedded proteins and protect them from extracellular milieu until they get disrupted either by the alkaline pH or the proteases secreted by cells, causing the polyhedra to unload their cargo. They can indeed be made by, *e.g.*, modifying the CPV genome with the addition of protein coding fragments. Co-expression of such chimeric proteins with wild-type or mutant polyhedrin in infected insect cells then leads to self-assembly of nanocontainers.^44^ Both, the capsid fragments^43^ as well as the polyhedrin proteins^45^ have been successfully used to drive incorporation of extrinsic proteins into polyhedra, however, the detailed molecular organization of the resulting superstructures is still largely unknown. The methods developed in our work can shed light on the principles underlying the structural organization of such nanocontainers, uncover new ways for cargo incorporation inside them, and rationalize the processes of their self-assembly and disassembly induced by the pH variation.

Elucidating the nature of Cypovirus capsid incorporation into the polyhedrin matrix, the role of VP5 proteins and the mechanisms driving polyhedra disassembly could stimulate development of new strategies for engineering virus polyhedra, resulting in an even broader spectrum of their applications.

## Methods

### Calculating electrostatic charges

In order to calculate the solvent accessible surface area of a molecule, we apply the algorithm described in ref. 46. The atoms of the molecule are modeled as spheres with non-covalent radii^18^ and a test sphere with radius 1.4 Å, corresponding to the radius of a water molecule, is rolled along the molecular surface. The resulting surface tracked by the center of the test sphere is by definition its SAS, whose area is used in the calculation of RSA.

Our simple coarse-grained electrostatic model considers each protein as a set of point charges that correspond to solvent-exposed ionizable amino acids.^31,32,34^ We calculate their partial charges using the Henderson–Hasselbalch equation:^28,38,39^1
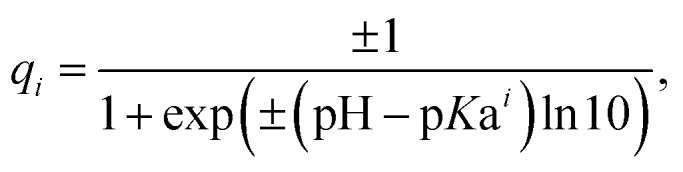
where the sign is defined by the type of AA: plus is used for basic amino acids (Arg, His, Lys) and minus for the acidic ones (Asp, Glu, Tyr). Values of the acid–base dissociation constant p*K*a^*i*^ of AAs are taken from ref. 47. The charges *q*_*i*_ are assumed to be localized in the centers of mass of the corresponding amino acids. We do not consider the protonation of Cys, because even though it has a thiol functional group that is susceptible for oxidation, the latter is reactive and often forms disulfide bonds.

### Energy of electrostatic interactions

The (free) energy of electrostatic interaction between two structural units is calculated within the standard Debye–Hückel approximation and reads:2
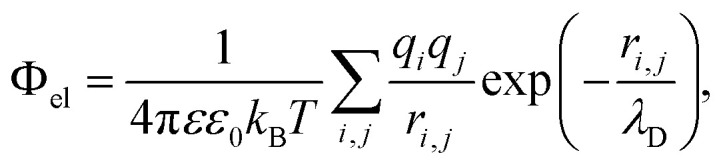
where indices *i*,*j* enumerate all solvent-exposed ionizable amino acids within first and second protein (or group of proteins, *e.g.* polyhedrin trimer and Cypovirus capsid) accordingly. Distances between corresponding point charges are denoted as *r*_*i*,*j*_. *T* = 300 K is the temperature, *k*_B_ is the Boltzmann constant, *ε* = 80 is dielectric permittivity of water and *ε*_0_ is permittivity of free space. Following ref. 32, 33 and 41, we estimate the Debye length *λ*_D_ at physiological conditions as:3
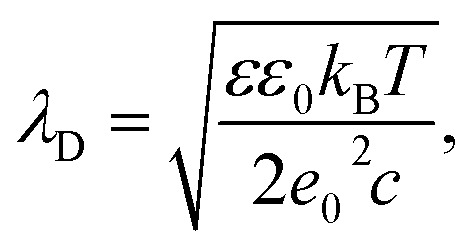
where monovalent salt concertation *c* is 100 mM and *e*_0_ is elementary charge, yielding the screening length *λ*_D_ = 1 nm.

## Author contributions

R. P. suggested to study virus polyhedra. O. K. proposed an initial structural model and developed it jointly with I. G. and S. R. I. G. and R. P. developed the electrostatic mechanism. The draft was written by O. K. and I. G. through contributions of S. R. and R. P. The project was managed by S. R.

## Conflicts of interest

There are no conflicts to declare.

## Supplementary Material

NA-005-D3NA00393K-s001

NA-005-D3NA00393K-s002

NA-005-D3NA00393K-s003
