# Supplementary material for: Integration of Cypoviruses into polyhedrin matrix
Source: Nanoscale Adv. 2023 Jul 16;5(16):4140–8. doi: 10.1039/d3na00393k (PMC10408579; doi:10.1039/d3na00393k)
Supplement: NA-005-D3NA00393K-s001 [file NA-005-D3NA00393K-s001.pdf]

Electronic Supplementary Material (ESI) for Nanoscale Advances.
This journal is © The Royal Society of Chemistry 2023

Electronic Supplementary Information for

Integration of Cypoviruses into polyhedrin matrix

Olga V. Konevtsova,[a] Ivan Yu. Golushko,[a] Rudolf Podgornik* [b,c,d] Sergei B. Rochal.**[a]

[a]Physics Faculty, Southern Federal University, Rostov-on-Don, Russia.
[b]School of Physical Sciences and Kavli Institute for Theoretical Sciences, University of Chinese Academy of Sciences, Beijing 100049, China.
[c]CAS Key Laboratory of Soft Matter Physics, Institute of Physics, Chinese Academy of Sciences, Beijing 100190, China.
[d]Wenzhou Institute of the University of Chinese Academy of Sciences, Wenzhou, Zhejiang 325000, China.
*Corresponding author. E-mail: rudolf.podgornik@ijs.si
**Corresponding author. E-mail: rochal_s@yahoo.fr

This PDF file includes:

Legends for ESI Movies 1 and 2

Other Supplementary Materials for this manuscript include:

ESI Movies 1 and 2

Movie 1. Interface between Cypovirus surface and polyhedrin cage. Polyhedrin trimers are shown in green, except for eight trimers (shown in red) occupying 3-fold axes of the superstructure and belonging to the vertices of the crystal fragment formed by 4x4x4 primitive polyhedrin cells. Triplets of the closest neighbors of the red trimers are shown in yellow. VP1 and VP5 proteins are shown in light purple and purple, respectively, 5-fold turrets assembled from VP3 are shown in grey.

Movie 2. Localization of NTP molecules in the vicinity of VP5 proteins. VP5 proteins are shown in purple, polyhedrin trimer is shown in crimson, ATP, GTP and CTP are shown in green, blue, and teal, respectively.
